# Supplementary material for: Deletion of wfs1 Impairs Oligodendrocyte Precursor Cells Dorsal Distribution and Myelination Through the wfs1-hmgcs1 Axis in Zebrafish
Source: Biology (Basel). 2026 Aug 16;15(16):1402. doi: 10.3390/biology15161402 (PMC13510151; doi:10.3390/biology15161402)
Supplement: Supplementary file 1 [file biology-15-01402-s001.zip › File S1-Transcriptome sequencing data and differentially expressed genes (DEGs) in wfs1-- zebrafish at 5 dpf/2.KEGG/wfs1KOvsWT/DOWN/src/dre00051.html]

dre00051


- dre:406339

- Down regulated genes

406339\_1(-1.38680349052238)

- dre:406339

- Down regulated genes

406339\_1(-1.38680349052238)

- dre:100007686

- Down regulated genes

100007686(-1.06018652619591)

- dre:568001

- Down regulated genes

568001\_2(-2.92834331323733)
- dre:561416

- Down regulated genes

561416(-4.80431336765849)

- dre:560753

- Down regulated genes

560753(-1.42485234049394)

Close
